# Elevated primary productivity triggered by mixing in the quasi-cul-de-sac Taiwan Strait during the NE monsoon

**DOI:** 10.1038/s41598-020-64580-6

**Published:** 2020-05-12

**Authors:** Ting-Hsuan Huang, Chen-Tung Arthur Chen, Yan Bai, Xianqiang He

**Affiliations:** 1 Department of Oceanography, National Sun Yat-sen University, Kaohsiung, Taiwan; 2 Ocean College, Zhejiang University, Zhoushan, China; 3 State Key Laboratory of Satellite Ocean Environment Dynamics, Second Institute of Oceanography, Ministry of Natural Resources, Hangzhou, China

**Keywords:** Physical oceanography, Marine chemistry

## Abstract

The Taiwan Strait (TS) connects two of the largest marginal seas in the world, namely the East China Sea (ECS) and the South China Sea (SCS). When the NE monsoon prevails, the fresh, nutrient-rich but P-limited China Coastal Current (CCC) flows southward. Yet, part of the CCC turns eastward after entering the TS and then turns back toward the ECS. In the southern TS, part of the salty, N-limited, northward TS current (TSC) in the eastern part of the strait turns westward and eventually returns to the SCS. That is, the TS acts like a quasi-cul-de-sac during the NE monsoon season. Based on 822 samples from 28 cruises, the highest Chl. a concentration occurs at a salinity around 32 even though the nutrient concentration is not the highest. Mixing the cold-fresh-eutrophic CCC water and the warm-salty-oligotrophic TSC water results in a more suitable condition for biological uptake in both the southern ECS and the northern SCS.

## Introduction

**Figure 1:**
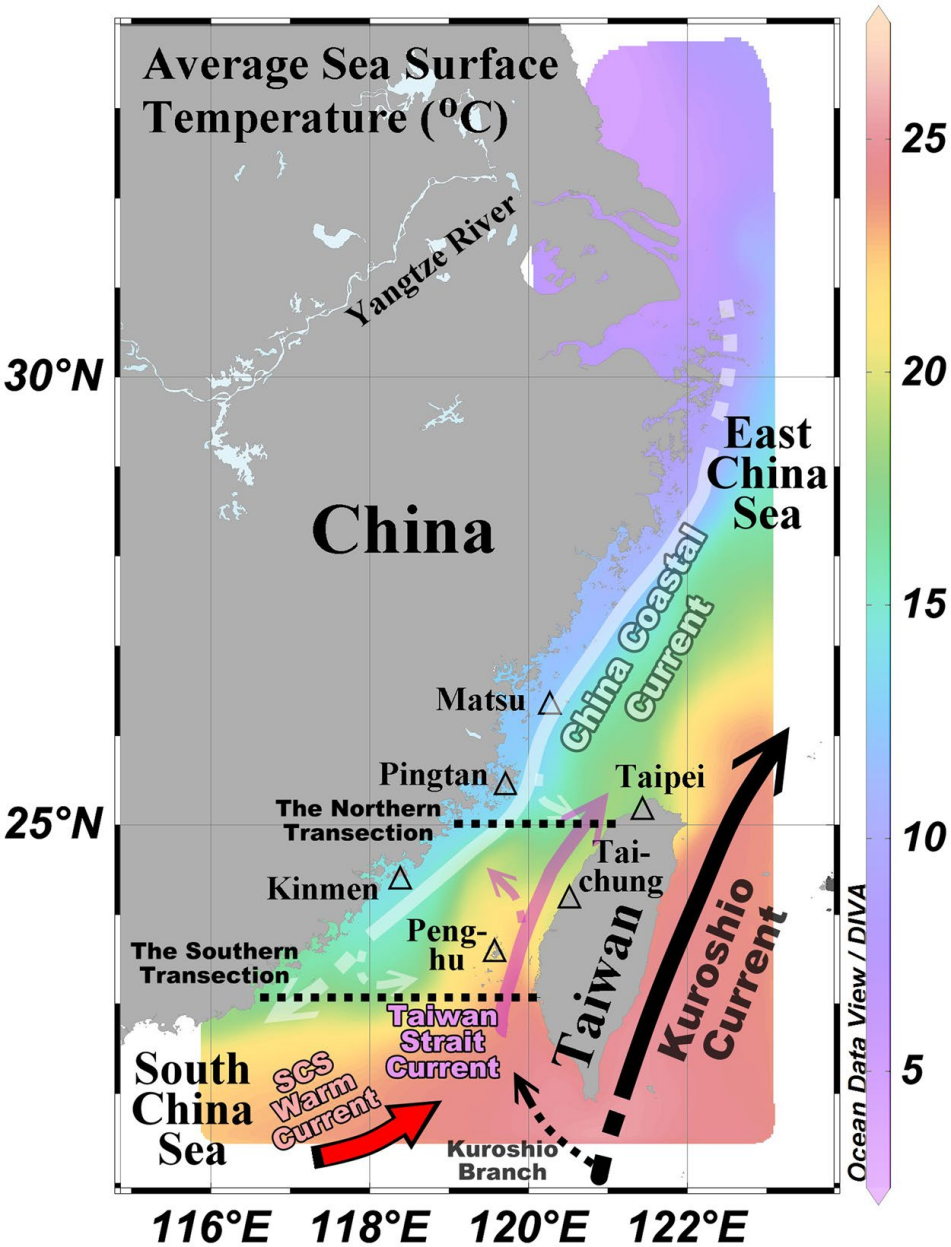
Averaged satellite sea surface temperature in February (2001–2010) and schematic currents in the study area. The white lines depict the China Coastal Current and its branch. The purple lines represent the Taiwan Strait Current and its branch. The black lines indicate the Kuroshio Current and its branch. The red line denotes the South China Sea Warm Current.

The Taiwan Strait (TS) is the only direct path between two of the largest marginal seas in the world, namely the nutrient-rich but largely phosphate (P)-limited East China Sea (ECS) and the oligotrophic and nitrogen-limited South China Sea (SCS)^1–4^. Exchanges of material through the TS affect the southern ECS and the northern SCS. Two currents coming from the south are the SCS Warm Current and the Kuroshio Branch through the Luzon Strait; they combine to produce the Taiwan Strait Current (TSC) (Fig. 1)^5,6^. The SW monsoon (May-Oct.) favors northward flow, which is also driven by the sea level difference, resulting in the highest water transport toward the north. On the other hand, in the NE monsoon season (Nov.-Apr.) the direction of the wind is opposite to the northward flow^7–9^.

The southward China Coastal Current (CCC) originates from the Yangtze River plume and incorporates other river plumes as it flows southward along the southeast coast of China Mainland (Fig. 1). The discharge of the Yangtze River in the NE monsoon season is low for the year (17430 ± 5515 m^3^ s^−1^). Yet, it still approximates the annual discharge of the Mississippi River^10^. The average temperature during the NE monsoon season is 4.5 ± 4.1 °C in the Yangtze River basin located between the subtropical and temperate zones^11^. Therefore, the southward, cold and fresh CCC occupies the western side of the TS while the northward, warm and salty seawater flows along the eastern strait. The location of the front between these two water masses varies with wind direction, wind speed, and river discharge^12,13^.

Three currents have been reported to cross horizontally the TS around 25°N, 24°N, and 23°N as a result of the interaction between wind in various directions and the topography (Fig. 1)^14–18^. The water masses differ in not only their physical but also their chemical and biological parameters. Notably, the TSC is one of the most important sources of phosphate for the P-limited ECS. On the other hand, the southward CCC contributes nitrate to the nitrogen-limited SCS^19,20^. This study focuses on how physical mixing between the CCC water and TSC water in the TS improves the biological uptake.

## Results and Discussion

**Figure 2:**
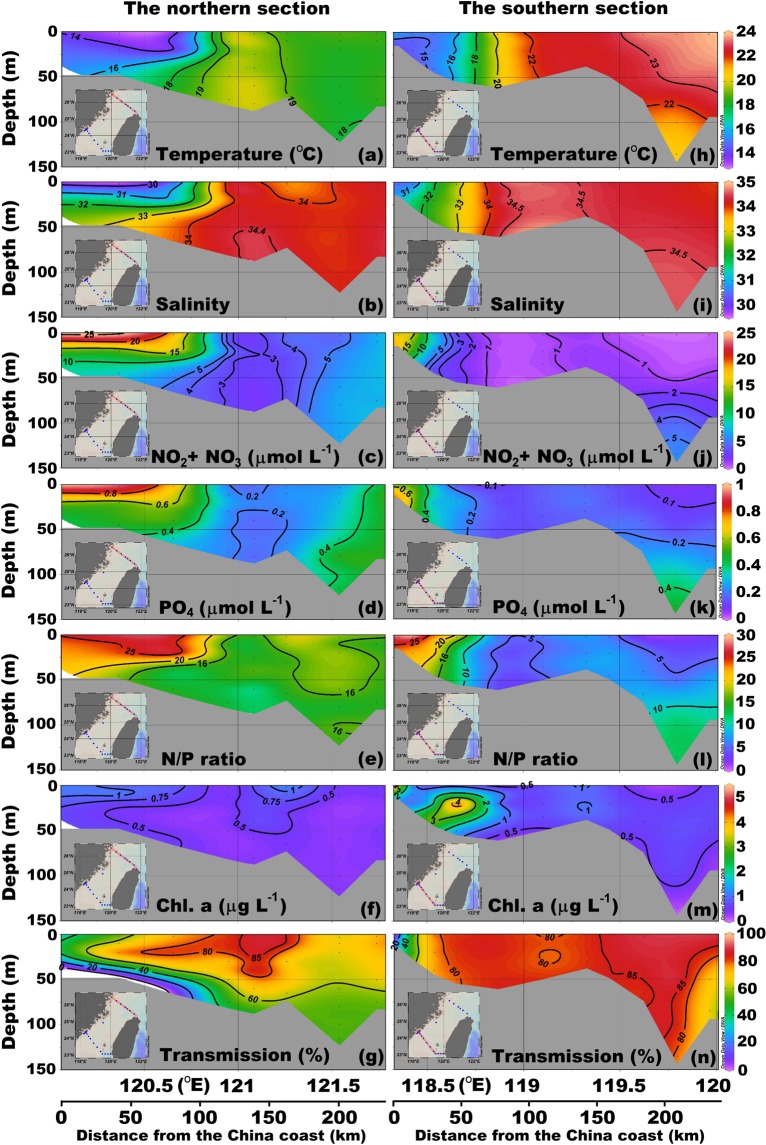
Cross sections of temperature, salinity, N, P, N/P ratio, Chl. a concentration, and transmission at the northern section (**a**–**g**) and the southern section (**h–n**) in February 11–13, 2009.

In the northern section of the TS, the temperature (13 °C) and salinity (S = 29) are lowest in the surface layer of the northwestern TS (e.g. during February 11-13, 2009; Fig. 2a,b). Cold and fresh water spreads around 70 km from the coast of China Mainland toward the middle of the strait. Notably, salinity in the surface layer of the middle TS is relatively low (S < 34; around 121.3°E). The temperature and salinity are highest in the northeastern TS. The NO_2_ + NO_3_ (N) as well as P concentrations and the N/P ratio are lowest in the area of high temperature and salinity (Fig. 2a–e). In the area of lowest temperature and salinity is found the highest N and P concentrations and the N/P ratio (Fig. 2a–e). The difference between the northwestern and northeastern TS is mainly due to the presence of at least two water masses.

Similarly, in the southern section the temperature and salinity are lowest in the western strait (Fig. 2h–i). Entire water columns are well mixed vertically, and the minimum temperature and salinity values increase, respectively, from 13 °C to 15 °C and from 29.7 to 30.6 along with the southward flow through both northern and southern sections (Fig. 2a,b,h,i). The region of the cold and fresh water (T < 15 °C, S < 31) is around 25 km from the coast of China Mainland, smaller than the extended distance in the northern section. The increase in temperature and salinity is a result of vertical and lateral mixing with the warm and salty seawater coming from the south. The temperature is highest in the surface layer of the southeastern strait. Seawater originated from the SCS flows along the western slope of the deepest region in the southern section, which is the Penghu channel (PC), toward the middle of the strait. The salinity is highest in this well-known upwelling area. As in the northern section, the N concentration and the N/P ratio are highest in the surface layer of the western strait, but the affected area is smaller in the southern section than in the northern section (Fig. 2c–e,j–l). The highest N (P) concentrations decrease from 25 µmol L^−1^ (0.8 µmol L^−1^) in the northern section to 15 µmol L^−1^ (0.6 µmol L^−1^) in the southern section. The seawater with low N concentration and low N/P ratio spreads from the southeastern strait to the border of the water with low salinity. The upwelled water from the PC is another source of N in the southern section. At the bottom of the PC, the temperature is relatively low whereas the salinity, N and P concentrations, and the N/P ratio are high. The N concentration is not relatively high in the middle of the strait although the strongest salinity signal is obtained from the upwelled water. This phenomenon may arise from biological consumption of N.

The two major water masses in the TS during the NE monsoon season are the CCC water and the TSC water. The low temperature (T ≈ 13–15 °C) and salinity (S ≈ 29–31) in the western parts of both the northern and the southern sections are characteristic of the CCC water (Fig. 2a,b,h,i). The high temperature (T ≈ 22–24 °C) and high salinity (S > 34) in the eastern part of the southern transection are associated with the TSC water (Fig. 2h,i). The southward CCC flows along the southeastern coast of China Mainland through the TS to the northern SCS. Meanwhile, part of the CCC turns toward the eastern TS and merges with the northward TSC (Fig. 1). Theoretically, the corresponding conditions of the seawater that is mixed from the CCC water and the TSC water are between those of these two water masses. Indeed, all of the physical and chemical values in the eastern part of the northern section are between those of these two water masses. There are two high Chl. a concentration (1.1 µg L^−1^) regions in the northern section. One is in the high N concentration area (around 120.3°E; 24.5µmol L^−1^), but the other is in the surface water of the eastern TS (around 121.25°E) with relatively low N concentration (4.1µmol L^−1^), one-sixth of the value from the CCC. This phenomenon may be associated partly with the changing temperature, N/P ratio, and light transmission of mixture seawater from the CCC and the TSC waters in the northwest TS (Fig. 2a–g). The highest Chl. a concentration in the southern section is in the mixed water area (5.4 µg L^−1^) rather than in the region of high N concentration (Fig. 2j–m; 3.6 µg L^−1^). Meanwhile, the highest Chl. a concentration with relatively low N concentration exists in the greater light transmission area around 121.25°E (Fig. 2g,n). By contrast, the low transmission limits the phytoplankton growth^21–23^.

**Figure 3:**
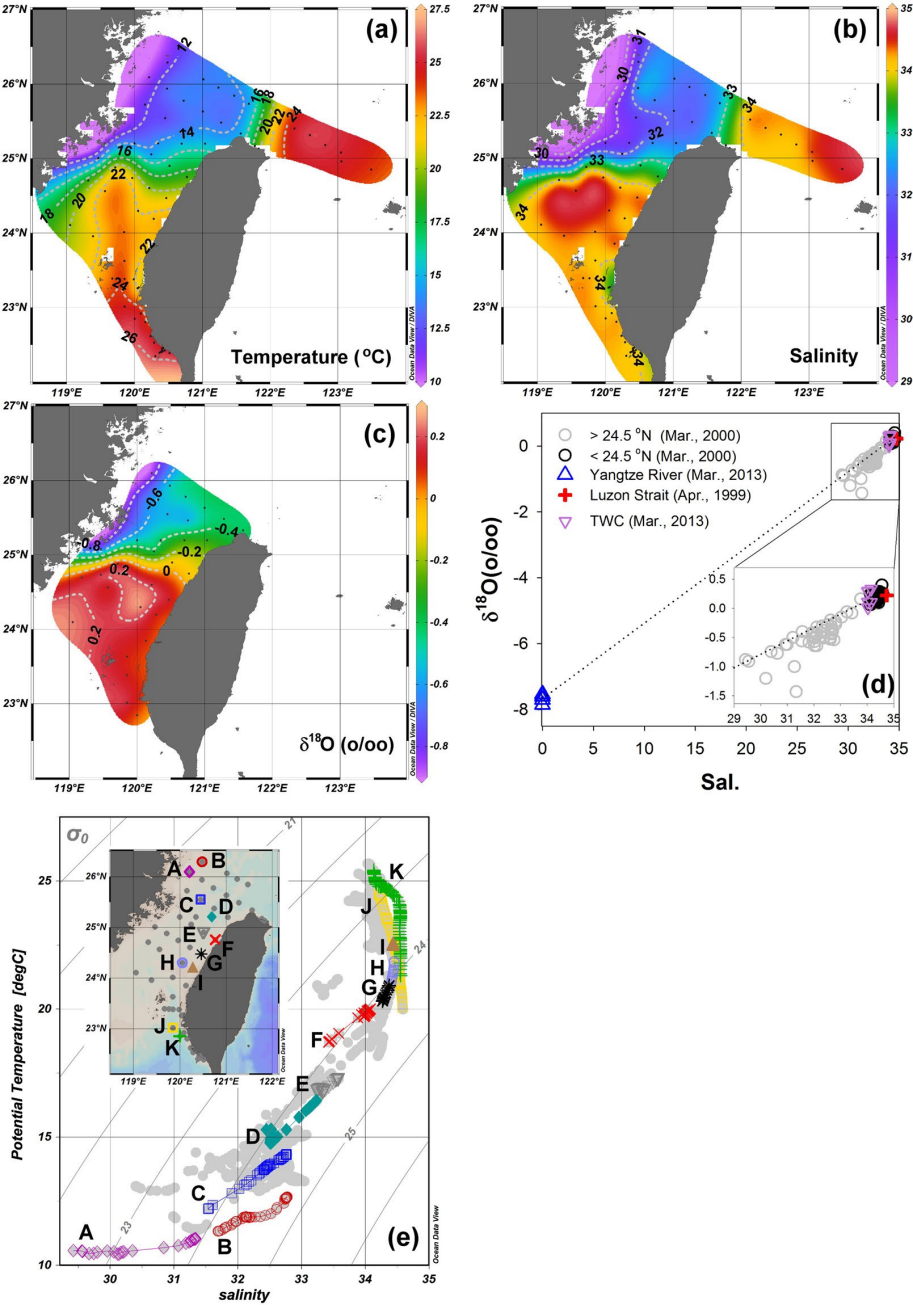
The distributions of surface (**a**) temperature (°C), (**b**) salinity, and c) δ^18^O in March 20–25, 2000. (**d**) The correlation between δ^18^O and salinity in the TS (circles), the Yangtze River (blue triangles), Luzon Strait (red crosses), and the southern ECS (red inverted triangles). (**e**) The potential temperature-salinity diagram during March 20–25, 2000; the map shows the sampling locations with corresponding symbols. The grey full circles depict the grey points on the map. The grey lines are isopycnals. The CTD data were adopted from ocean data bank (www.odb.ntu.tw). The δ^18^O were collected from Chen and Sheu^26^, Lian *et al*.,^25^, and Lin^27^.

The potential temperature-salinity diagram and stable isotopes have been adopted for determining water masses. The surface distribution of temperature, salinity, and δ^18^O values increase from the northwestern TS to the southeastern TS (Fig. 3a–c). The ratio of stable isotopes ^18^O and ^16^O is an indicator of water cycle processes. The lower vapor pressure of H_2_^18^O than H_2_^16^O is the main factor for isotope fractionation during evaporation and condensation resulting in higher proportions of ^18^O in seawater^24^. The cold, fresh, and low-δ^18^O value seawater (the CCC water) originates from the Yangtze River^25,26^. The warm, salty, and high-δ^18^O value seawater (the TSC water) is derived from the seawater of the Luzon Strait (Fig. 3a–d)^27^. The δ^18^O values of the seawater in the northern TS (>24.5°N, the grey circles in Fig. 3d) are between the values of the Yangtze River and the seawater from the Luzon Strait. This phenomena suggests that the seawater in the northern TS is resulted from the mixing of two water masses, then flows toward the southern ECS. Additionally, the potential temperature-salinity diagram provides more detail about the mixing process. The pattern between the lowest salinity (purple diamonds in Fig. 3e) and the highest salinity (green crosses in Fig. 3e) is not a straight line, and represents at least a three-water mass mixing. On the other hand, in the middle (locations C to H, Fig. 3e) is a straight line from the surface (lower temperature and salinity) to the bottom (higher temperature and salinity) for waters shallower than 150 m. This result suggests the processes reflecting both vertical and lateral mixing. The exact mixing mechanism has not been resolved fully even though some published articles provided factors related to the transitory cross-strait currents^14,16^. The drifter data also support the cross-strait currents existing in the TS, and data from the drifter also recorded the varied water temperature due to the mixing process (see Supplementary Information Fig. S1). The distribution of the long-term averaged sea surface temperature from satellites also implies a mixing process (see the green color in Fig. 1 occupying half of the northern TS and one-third of the southern TS).

**Figure 4:**
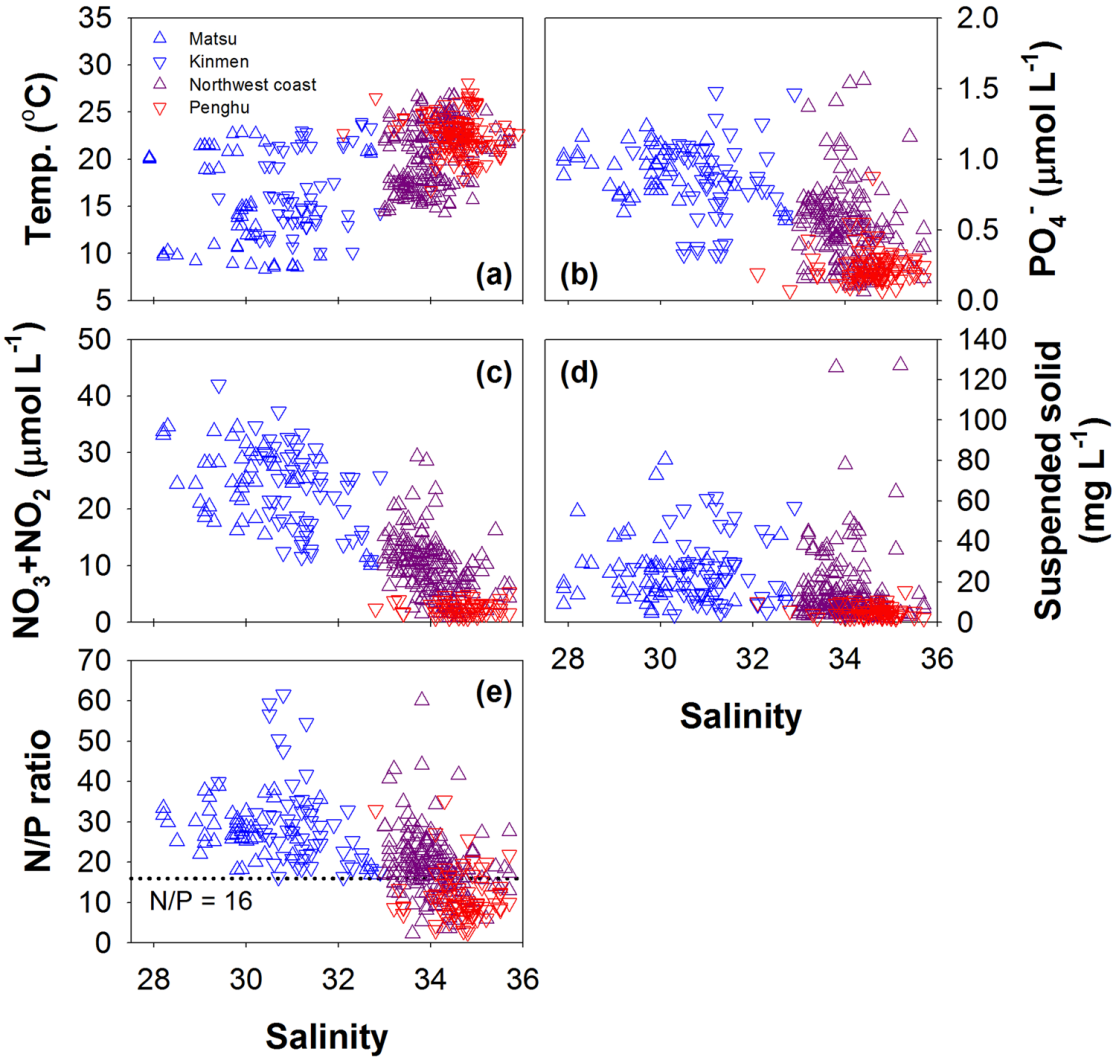
Correlations between (**a**) temperature, (**b**) P concentration, (**c**) N concentration, (**d**) suspended solid concentration as well as (**e**) N/P ratio and salinity, respectively.

Since the cruises provided information only during the sampling periods, additional measured surface data close to the coasts were obtained from the Environmental Protection Agency (EPA) of Taiwan. Overall, the temperature increases with salinity, but the nutrient concentrations decrease with salinity (Fig. 4a–c). The seawaters around Matsu Is. and Kinmen Is. are cold and fresh and characterized by high nutrient concentrations, just like the CCC (blue symbols in Fig. 4). The seawater around Penghu Is. exhibits high temperature and salinity but low nutrient concentrations similar to the feature of the TSC water (red symbol in Fig. 4). The physical and chemical data for the northwest Taiwan coastal seawater (purple triangle in Fig. 4) are between those for the seawater around Penghu Is. and for the seawater in the western TS around Matsu Is. and Kinmen Is., consistent with results obtained during the cruises (Figs. 2, 3). The concentrations of suspended solids are related to the light transmission. If there are more suspended solids, there is lower transmitted light. Generally, the suspended solids are high in the CCC water (blue symbols in Fig. 4d) and the values are low in the TWC water (red symbol in Fig. 4d). Interestingly, the N/P ratios are near the Redfield ratio (N/P = 16) in the mixed seawater region (Fig. 4e).

**Figure 5:**
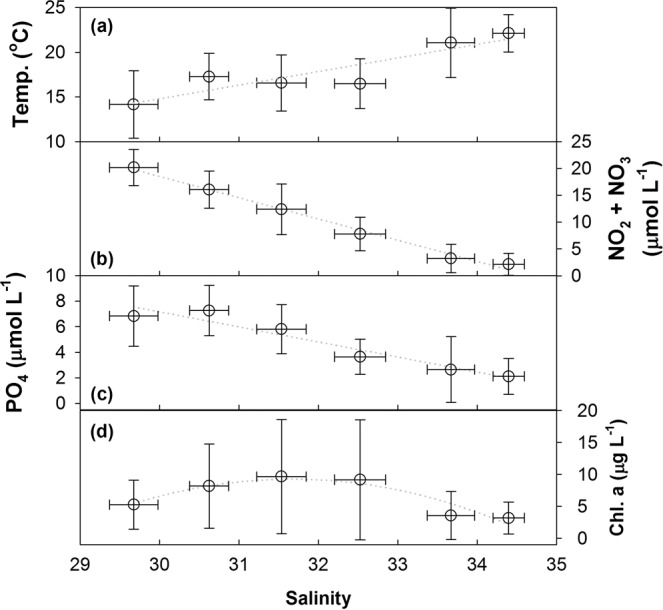
Averaged (**a**) temperature, (**b**) N concentration, (**c**) P concentration, and (**d**) Chl. a concentration in different salinity ranges. The sample numbers of each group is as follows: 29 ≤ S < 30: 6; 30 ≤ S < 31: 47; 31 ≤ S < 32: 57; 32 ≤ S < 33: 50; 33 ≤ S < 34: 147; 34 ≤ S < 35: 515.

The 822 samples with different sampling depths from 28 cruises in the TS are categorized into six groups according to the ranges of salinity (Fig. 5 and Supplementary Information Fig. 2). The temperature increases with the salinity. However, the temperature decreases slightly between the salinity range of 30 to 33 (Fig. 5a). The nutrient concentrations clearly decrease with salinity (Fig. 5b,c). Interestingly, the Chl. a concentrations show a different pattern. The higher Chl. a concentrations are in the middle salinity range resulting from the mixing between two water masses (Fig. 5d). The mixed seawater provides a more suitable environment for the phytoplankton growth such as higher light transmission and warmer water temperature compared with the CCC even though the nutrient concentrations are lower.

**Figure 6:**
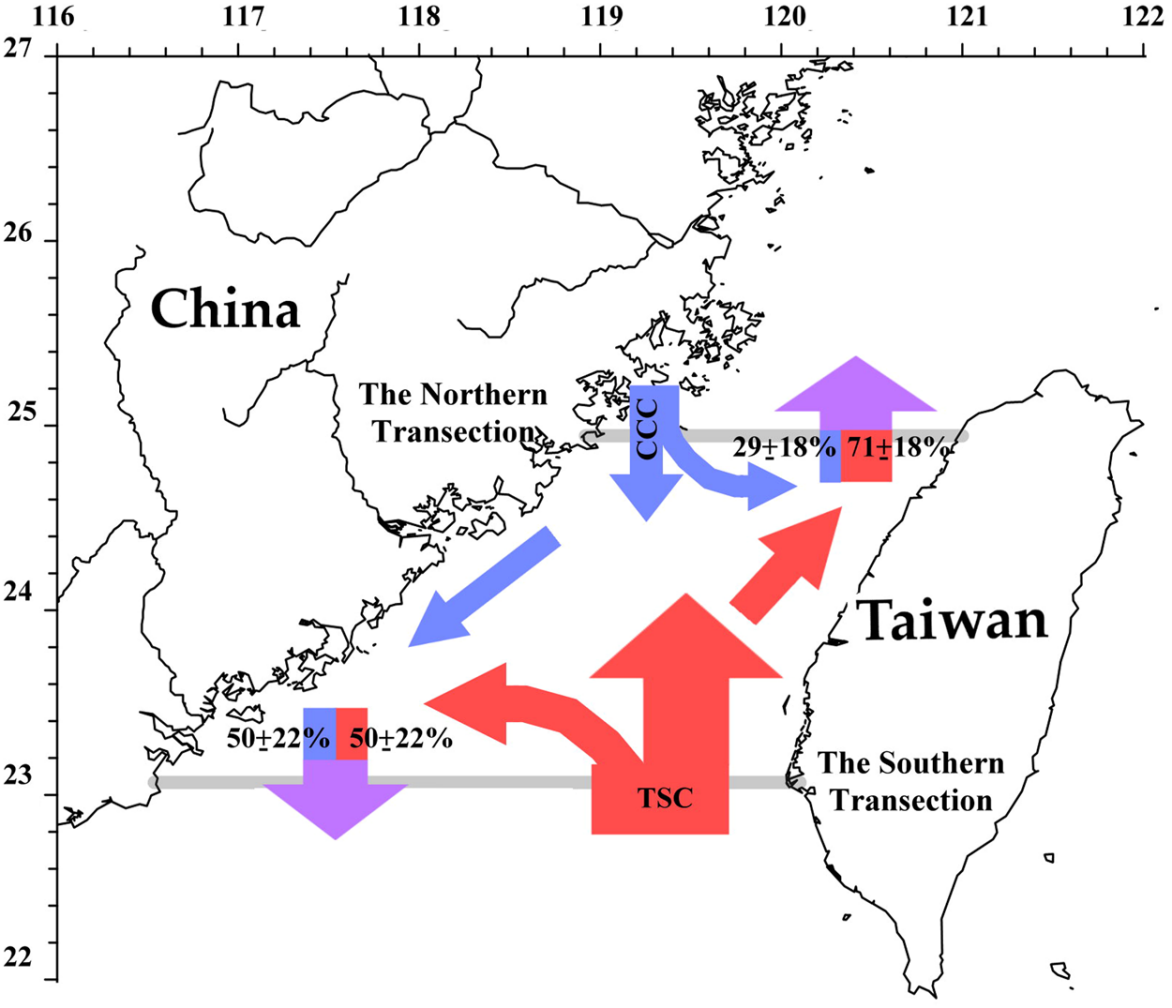
Schematics for water mass compositions and currents. The blue, red, and purple areas represent the China Coastal Current (CCC), the Taiwan Strait Current (TWC), and mixture waters from the CCC water and TSC water. The grey lines depict the northern and southern transections.

Based on the published articles, there are two admixtures in the TS reflected by 1) the northward TSC mixed with the southward-turned-northward CCC, and 2) the northward-turned-southward TSC mixed with the southward CCC (Fig. 1)^8,16^. To estimate the mixing ratio of the water masses, the Hybrid Coordinate Ocean Model (HYCOM) data and empirical formulas were used. Modeled water salinity and nutrient transport data along 23.04°N (the southern transection) and 24.96°N (the northern transection) were calculated and separated into northward transport data and southward transport data for each transection (Fig. 1). Then, water mass percentages were estimated from the modeled salinities of the two major water masses. Here, the average salinity of the southward flow in the northern transection is taken as the salinity for the CCC water, and the average salinity of the northward flow in the southern transection is taken as the salinity for the TSC water. For instance, the salinity values of the CCC water, TSC water, and the northwardly flowing water in the northern transection are 33.89, 34.37, and 34.22, respectively. The northward flowing water comprises 31% CCC water and 69% TSC water. Overall, during the NE monsoon season from 1993 to 2012, 29 ± 18% of the northward flowing water is derived from the CCC water after the latter turns eastward. The southward flowing water is composed of 50 ± 23% of TSC, after the latter makes an eastward turn. The remaining percentage of the southward flowing water is derived from the CCC (Fig. 6). The N/P ratios of the CCC water and the TSC water are 20.3 ± 7.4 and 9.4 ± 1.1, respectively. The average N/P ratios of the northward flow in the northern transection and the southward flow in the southern transection are 15.2 ± 12.1 and 13.0 ± 1.8, respectively. That is, the N/P ratios of the mixed waters are closer to the Redfield ratio compared to the CCC and TSC waters.

## Summary

The TSC is one of the most important sources of phosphate for the ECS. The results from this study suggest that in the northern TS the northward TSC mixes with the southward CCC after part of it turns and becomes the northward Taiwan Warm Current in the NE monsoon period. Similarly, in the southern TS part of the northward TSC merges with the remaining southward CCC to become the southward flow. Wind and the terrain, which create a quasi-cul-de-sac environment, contribute to mixing. Approximately 50% of the southward flow that passes through the TS to the northern SCS originates from the CCC water, and around 79% of the northward flow derived from the TSC water reaches the ECS. Chlorophyll a concentrations are enhanced when there is mixing.

## Materials and Method

This study employs chemical composition measurements which were collected in the TS between 1997 to 2013, over the span of 28 cruises which all occurred during the NE monsoon season from November to April (see Supplementary Information Fig. 2).

The measured chemical data are nitrate plus nitrite (N) and phosphate (P). The total N concentration was obtained using the pink azo dye method and a flow injection analyzer with an on-line Cd-coil yielding a precision of approximately ±1% at 36 μmol L^−1^ and ±3% at 1 μmol L^−1^. The concentration of P was determined by the themolybdenum blue method using a flow injection analysis with a precision of approximately 0.5% at 2.9 μmol L^−1^ and 3% at 0.1 μmol L^−1^. The Chl. a concentration was measured by a Turner Designs model 10-AU fluorometer and samples were extracted with 90% acetone from a 0.45 μm pore size Millipore filter^28^.

The drifter data were collected from the Global Drifter Program (https://www.aoml.noaa.gov/phod/gdp/). The sampling stations from the Environmental Water Quality database of the Environmental Protection Administration (EPA) of Taiwan (2002–2014) were located in Matsu Island, Kinmen Island, Penghu Island, and the coastal area of northwest Taiwan from Taipei to Taichung (Fig. 1 and Supplementary Information Fig. 2). The monthly discharge data of the Yangtze River (1993–2012) at the Datong station were obtained from the annual ChangJiang Sediment Bulletin (published on http://www.cjh.com.cn). Satellite sea surface temperatures for every February from 2001 to 2010 were obtained from the European Space Agency (ESA Sea Surface Temperature Climate Change Initiative^29^).

Empirical formulae were then applied to combine these direct measurements of shipboard CTD values and chemical data along with satellite measurements and modeled data for salinity, temperature, and current speed (HYCOM). Empirical formulae for the N and P concentrations were driven as second-order polynomial regression equations from CTD salinity, CTD temperature, and measured chemical results. Further, we adopted the daily HYCOM salinity and temperature to calculate daily N and P concentrations with empirical formulae. These formulas allow us to expand these sparse direct measurements into approximated full time series profiles, as described in Huang *et al*.^20^. The transported water amounts were estimated from modeled salinity and current speed. The average salinity and temperature differences between modeled and CTD-measured data are 0.10 ± 0.12 and −0.3 ± 3.7 °C, respectively (see Supplementary Fig. S3a,b). The adjusted determination coefficients between the fitted and the measured N (P) concentration is 0.92 (0.77, see Supplementary Fig. S4a,b). After the empirical formulas were validated, we used the HYCOM salinity and temperature to estimate the model-driven N and P concentrations as well as N/P ratios. The average differences in N, P, and N/P ratio between model-driven and measured data are −1.4 ± 3.7 μmol L^−1^, −0.03 ± 0.16 μmol L^−1^, and −1.5 ± 7.5, respectively (see Supplementary Fig. S3c-e).

## Supplementary information

Supplementary Materials.

## Data Availability

The cruise data are available from the ocean data bank (www.odb.ntu.edu.tw).
