# Supplementary material for: Elevated primary productivity triggered by mixing in the quasi-cul-de-sac Taiwan Strait during the NE monsoon
Source: Sci Rep. 2020 May 12;10:7846. doi: 10.1038/s41598-020-64580-6 (PMC7217951; doi:10.1038/s41598-020-64580-6)
Supplement: Supplementary file 1 — Supplementary Materials. [file 41598_2020_64580_MOESM1_ESM.docx]

**Supplementary Materials for**

**Elevated primary productivity triggered by mixing in the quasi-cul-de-sac Taiwan Strait during the NE monsoon**

Ting-Hsuan Huang, Chen-Tung Arthur Chen, Yan Bai, Xianqiang He

Correspondence to: ctchen@mail.nsysu.edu.tw


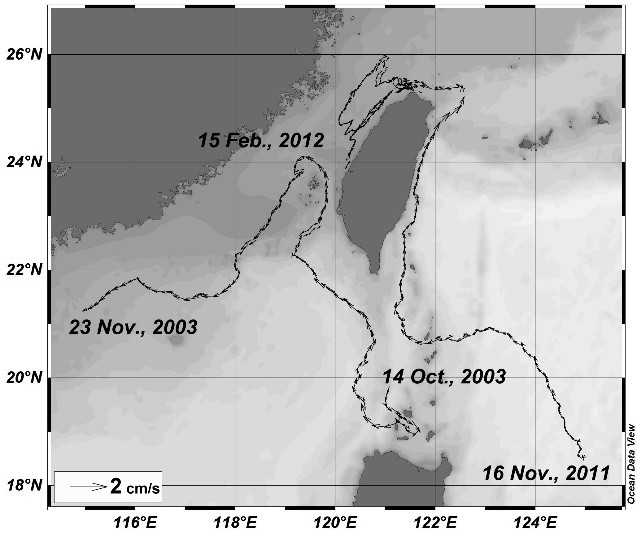

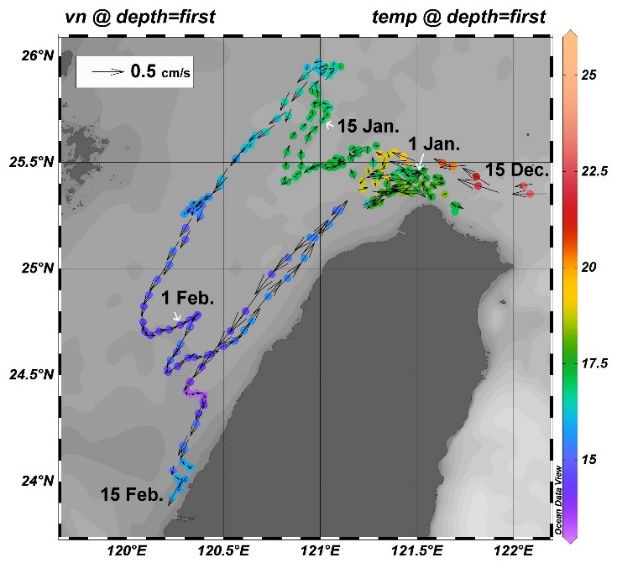


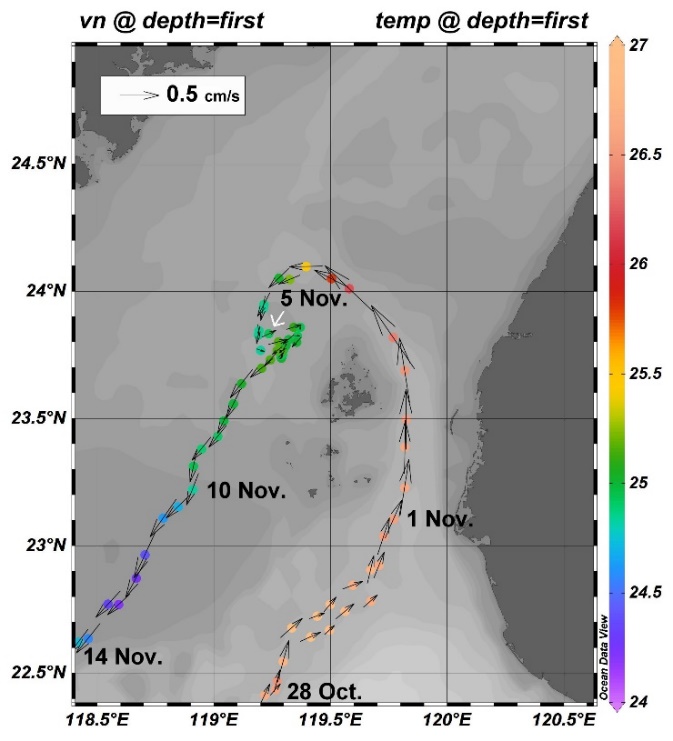


Figure S1 Transport routes for drifters. The arrows represent the speed and direction. The color scale is surface temperature (°C).


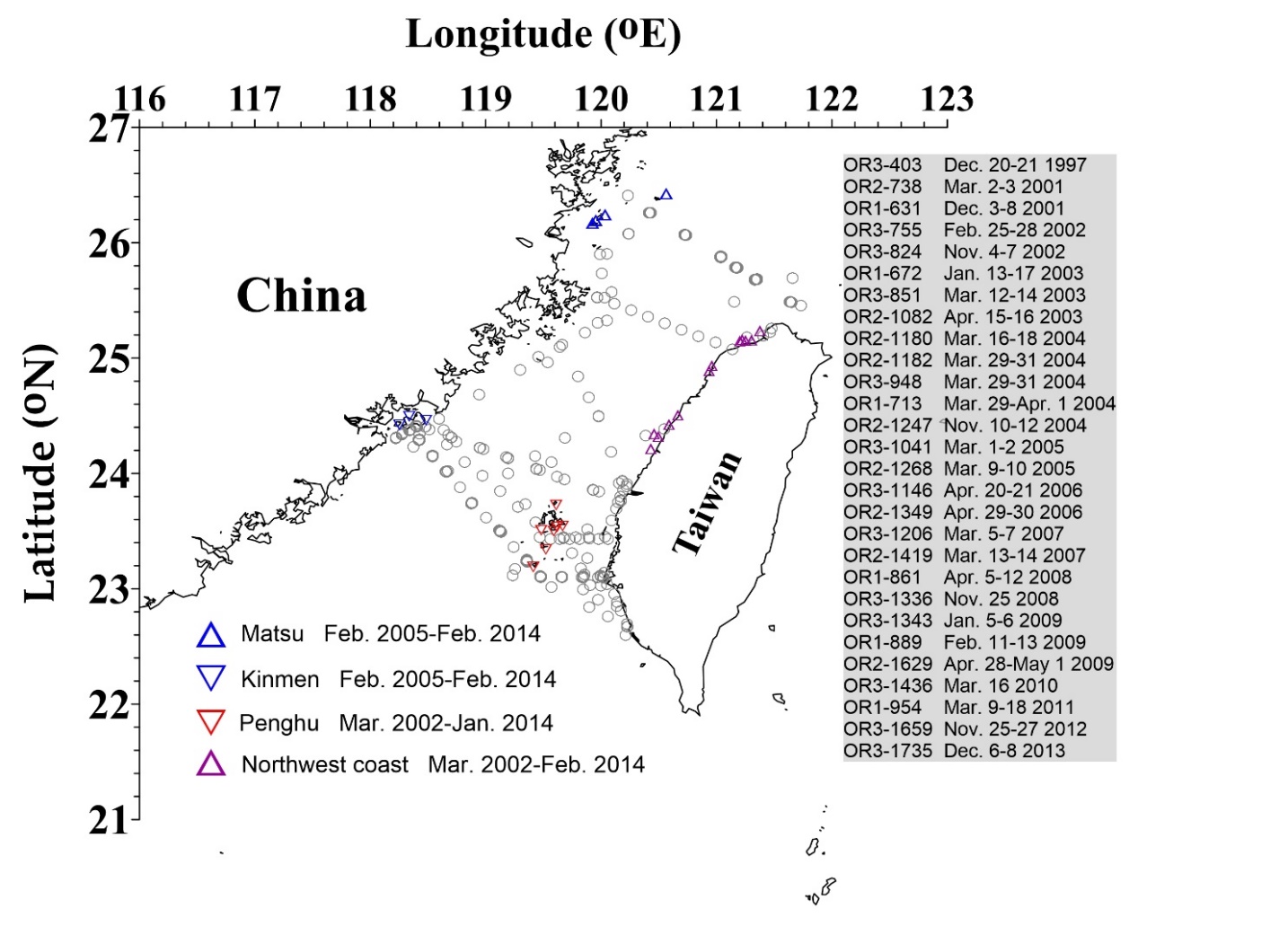


Figure S2 Sample locations from C.T.A.C’s 28 cruises (grey circles) and the EPA of Taiwan (triangles).


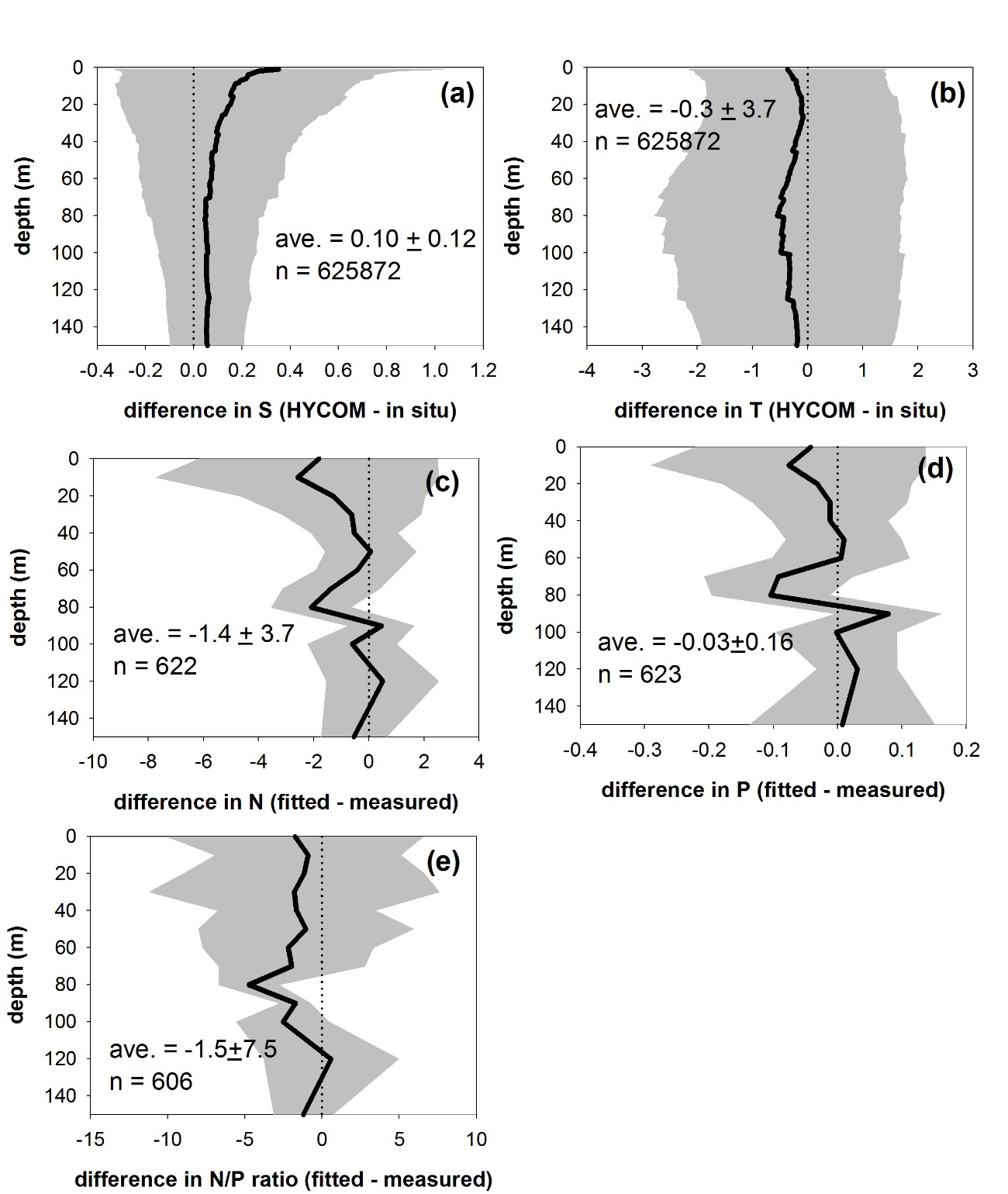


Figure S3 Differences in a) salinity, b) temperature, c) N concentration, d) P concentration, and N/P ratio between HYCOM-driven results and measured values at various depths during November to April. Black lines represent averaged difference and grey area indicates error range. The HYCOM driven N and P concentrations were calculated using empirical formulas from Huang et al. (2019) and HYCOM salinity and temperature.


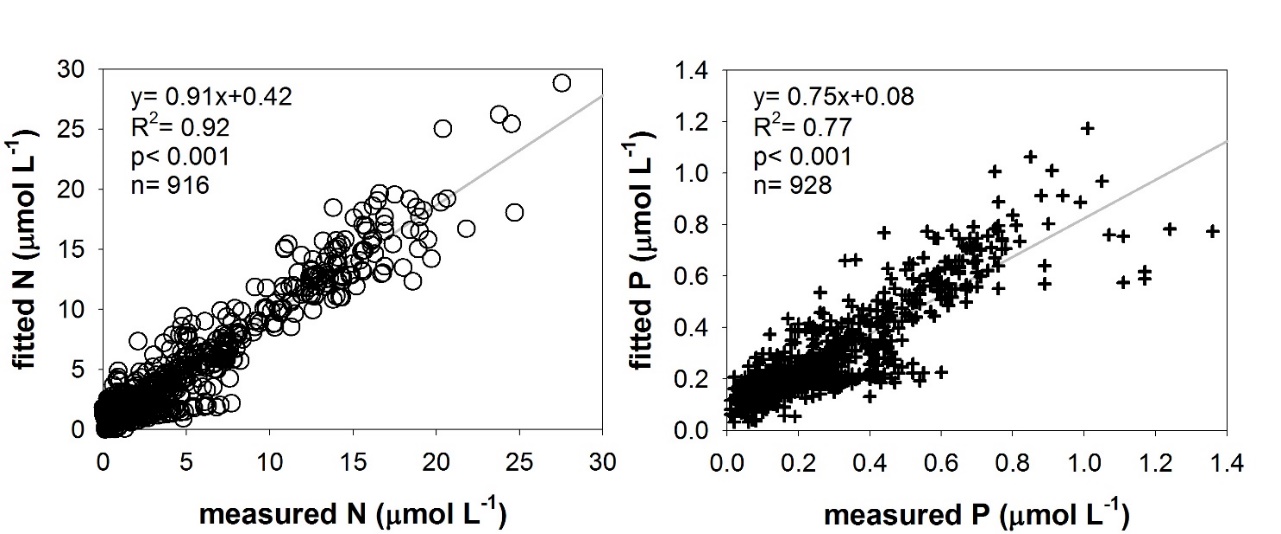


Figure S4 Correlations between fitted and measured concentrations of a) N and b) P. The fitted concentrations were calculated using empirical formulas from Huang et al. (2019) and CTD salinity and temperature.
